# Are senior high school students in Ghana meeting WHO’s recommended level of physical activity? Evidence from the 2012 Global School-based Student Health Survey Data

**DOI:** 10.1371/journal.pone.0229012

**Published:** 2020-02-12

**Authors:** Abdul-Aziz Seidu, Bright Opoku Ahinkorah, Ebenezer Agbaglo, Eugene Kofuor Maafo Darteh, Edward Kwabena Ameyaw, Eugene Budu, Hawa Iddrisu

**Affiliations:** 1 Department of Population and Health, University of Cape Coast, Cape Coast, Ghana; 2 The Australian Centre for Public and Population Health Research (ACPPHR), Faculty of Health, University of Technology Sydney, Sydney, Australia; 3 Department of English, University of Cape Coast, Cape Coast, Ghana; 4 Ghana Education Service, Techiman Municipal, Bono East Region, Techiman, Ghana; Griffith University, AUSTRALIA

## Abstract

**Introduction:**

Physical activity (PA) has both short- and long-term importance. In this study we sought to assess the prevalence and correlates of PA among 1,542 Senior High School (SHS) students.

**Methods:**

A cross-sectional study was conducted in Ghana among SHS students using the 2012 version of the Ghana Global School-based Student Health Survey (GSHS) data, which utilised two-stage cluster sampling technique. The population for the study comprised SHS students. The outcome variable was physical activity. The data were analysed using STATA version 14.2 for Mac OS. Both bivariate and multivariate analyses were employed. At the bivariate level, Pearson chi-square test between each independent variable and PA was conducted and the level of statistical significance was set at 5%. All the significant variables from the chi-square test were selected for the multivariate analysis. In the multivariate analysis, Poisson regression with robust variance was performed to estimate crude and adjusted prevalence ratios (APR).

**Results:**

It was found that 25.0% (29.0% males and 21.9% females) of SHS students were physically active. Female students (APR = 0.78, 95% CI = 0.65, 0.94), students in SHS 2 (APR = 0.76, 95% CI = 0.577, 0.941) and SHS3 (APR = 0.79, 95% CI = 0.63, 0.93), and those who went hungry (APR = 0.77, 95% CI = 0.65, 0.92) were less likely to be physically active compared to males, those in SHS1 and those who did not go hungry respectively. On the other hand, students who actively commuted to school (APR = 2.40, 95% CI = 1.72, 2.42) and got support from their peers were more likely to be physically active (APR = 1.62, 95% CI = 1.09–2.41).

**Conclusion:**

Only a quarter of SHS students who participated in the 2012 version of the GSHS met the WHO’s recommended level of physical activity. Sex, grade/form and experience of hunger are associated with physical activity. Physical activity is a major component of any health promotion program. Policies and programmes targeting improvement in physical activity among SHS students should take these associated factors into consideration.

## Introduction

Activities involving body movement triggered by skeletal muscles are collectively referred to as physical activity. These activities (for instance, travelling) usually involve energy expenditure [1, 2]. The World Health Organisation (WHO) recommends that children and adolescents should have not less than 60 min of daily physical activity of moderate-to-vigorous intensity or as being active for not less than 60 min on 5 days per week [3].The WHO additionally notes that physical activity, when done regularly by adolescents, comes with health benefits (such as, cardiovascular and metabolic health), while physical inactivity may result in ill health [3]. Less fat mass [4] as well as the probability of becoming a healthy adult [5] has also been noted as benefits of physical activity.

Despite the significance of physical activity, research has shown that physical inactivity—not meeting WHO recommendation—among adolescents all over the world is on the rise [6–8]. Concerns have been raised about physical inactivity among adolescents because of the likelihood of them maintaining similar levels of physical inactivity into adulthood, and this may have negative effects on their health [9]. As a result, there has been a global call for action among nations to collectively work towards improving knowledge on physical activity [10]. This has led to an upsurge of research on the subject, with the focus on factors associated with physical activity among adolescents and students [11, 12], motives for physical activity among adolescents [1, 13, 14], as well as frequency of adolescents’ involvement in physical activity [2, 15, 16]. Additionally, factors influencing physical activity have been documented by some previous studies [2, 17, 18].

A review of the literature suggests that previous studies on physical activity focused largely on Europe [14, 19, 20], and American countries such as Brazil [11, 16, 17], United States of America [9], and Peru [2], with few studies in Africa. Generally, in the context of Africa, Nigeria [12] and South Africa [21] have been the focus of the few previous studies of this kind. Peltzer [22] also investigated the phenomenon from a cross-country perspective while Guthold et al. [23] investigated the phenomenon from a global perspective. In the Ghanaian context, physical activity only formed an insignificant part of some previous studies with a broader focus [23–28]. In effect, such studies do not present a panoramic view of the determinants of physical activity among Senior High School (SHS) students in Ghana. This study, therefore, aims at addressing this knowledge gap by investigating the prevalence and correlates of physical activity among Ghanaian SHS students.

## Materials and methods

### Study design and participants

We used data from the 2012 version of Ghana Global School-based Student Health Survey (GSHS) [29]. The GSHS was conducted by the WHO in collaboration with Disease Control and Prevention (CDC), Middle Tennessee State University, and the Ghana Education Service (GES). The first one was in 2007. The GSHS uses a validated questionnaire across the world and has been in inception since 2003. The GSHS primarily aims at in-school adolescents. It includes validated survey items selected from ten core modules, namely “nutrition”, “physical activity”, “hygiene”, “mental health”, “alcohol use”, “tobacco use”, “drug use”, “sexual behaviours”, “violence/injury”, and “protective factors” [29, 30].

The data were collected using a cross-sectional survey design for WHO countries and aimed at examining behavioural risk factors and protective factors in several domains. Data collection was done by the use of close-ended structured questionnaires administered to the students. The detailed description of how the data were collected can be found on the WHO website (see WHO [29]). The students were in grades/forms (SHS 1–4), which are typically attended by students aged 13–17. However, the study was not limited to only students aged 13–17 but considered all the students from SHS 1–4, irrespective of age. The students were sampled from selected SHSs in all the then ten (10) administrative regions of Ghana. The study employed a two-stage cluster sampling design to select 25 SHSs to represent all the then 10 regions in Ghana. What informed the choice of the selection of the schools at the first stage of the sampling was based on a probability proportional to the size of enrolment. The second stage was characterised by a random sampling technique which was used to select the classes in each school. This subsequently gave each and every student an equal probability of participating in the study.

Computer-scannable answer sheets were used to by the students to self-reported their responses to each of the questions posed. The response rate recorded at the school level was (96%) while the students response rate was 74%, making the overall response rate was 71%. The total number of students who took part in the survey was 1,984. Of these more than half (1,065 [53.7%]) were males while 908 (45.7%) were females. Less than (0.6%,n = 11) one percent of the variables had missing data [30 p.76]. Following some previous studies [10, 28], we excluded respondents with missing data from the study and this yielded a sample of 1,542 respondents for our analysis. The design, implementation and reporting of results followed the Strengthening the Reporting of Observational Studies in Epidemiology (STROBE) checklist for cross-sectional studies (supplementary file, S1 Table).

### Measurement of variables

#### Ascertainment of outcome variable

Physical activity was the main outcome of interest. As evidenced in several previous studies [2, 10, 11, 15, 17, 22, 28, 31], physical activity was measured with a single self-reported question. The question was: “During the past 7 days, on how many days were you physically active for a total of at least 60 minutes per day?” and has been operationalized as 5 days per week. The options provided ranged from zero to seven days. Based on the WHO [3] recommendation that there should be at least 60 minutes of physical activity each day for five days or more and drawing from previous studies [2, 10, 11, 15, 17, 22,23, 28, 31], the SHS students were grouped into two. Thus, those who met the recommendation for engaging in 5 days or more physical activity in the last week preceding the survey were categorised as “sufficiently physically active” while those who did not meet this standard were grouped as “physically inactive”.

#### Ascertainment of independent variables

Sixteen independent variables were used to assess their association with physical activity among the students. These were broadly grouped into socio-demographic variables, substance and alcohol use and other social behaviour, and socio-familiar support variables. The socio-demographic variables included were age, sex and hunger–a proxy measure of socio-economic status. The other variables included fruits and vegetables consumption, tobacco and alcohol use, bullying victimisation, sedentary behaviour, active commuting to school, physical education (PE), class attendance, peer support, parents checking homework (parental supervision), parents understanding adolescents’ problems (parental connectedness), and knowing what adolescents do at their free time (parental or guardian bonding). The variables were chosen based on two principal reasons: first, their availability in the GSHS dataset and second, conclusions previous studies have drawn about them as having association with physical activity [2, 10, 11, 15, 17, 22, 28, 31]. Some of the variables were dichotomised in consonance with the GSHS methodology and previous studies [32]. Detailed description of the variables, the questions they were derived from, and how they were recoded are shown in supplementary file, S2 Table.

### Statistical analysis

The data were analysed using STATA version 14.2 for Mac OS. A pie chart was used to present results on the prevalence of physical activity using frequencies and percentages. Aside this, both bivariate and multivariate analyses were employed. At the bivariate level, Pearson chi-square test between each independent variable and physical activity was conducted and the level of statistical significance was set at 5% (see Table 1). All the significant variables from the chi-square test were selected for the multivariate analysis. In the multivariate analysis, Poisson regression with robust variance was performed to estimate crude and adjusted prevalence ratios (PR) of physical activity as recommended by Barros and Hirakata and Tamhane et al.[33,34]. Specifically, the multivariate analysis followed a conceptual two hierarchical models that determined the order of entry of variables into each model to control for potential confounding factors (see Table 2). The first level was an incomplete model with demographic and socio-demographic variables (sex, grade, and hunger). The second, a complete model, used the adolescents’ active commuting to school, number of close friends, bullying victimization and parental bonding variables to ascertain how the socio-demographic as well as the additional variables will interact to predict physical activity participation. Multicollinearity was checked with variance inflation factor and there was no evidence of multicollinearity among the variables since the maximum VIF was 2.5 and a VIF below 10 is considered acceptable. All frequency distributions were weighted while the survey command (svy) in STATA was used to adjust for the complex sampling structure of the data in the regression analyses to enable generalization of results to the eligible population, SHS students.

**Table 1:** Distribution of the sample according to level of physical activity.

Variables	N = 1,542			95% CI	P-value
	Frequency	Percentage	Active		
**Sex**					**0.000
Male	785	50.9	29.0	26.1–32.2	
Female	757	49.1	21.9	18.3–24.4	
**Age**					0.233
12–17	708	45.9	35.0	23.8–30.2	
18 and above	834	54.1	25.5	21.4–27.4	
**Grade**					
SHS1	397	25.7	28.9	24.6–32.7	**0.002
SHS2	404	26.3	20.1	15.9–25.0	
SHS3	415	26.9	22.4	18.9–26.3	
SHS4	326	21.2	31.5	26.4–37.1	
**Socio-economic Status (hunger)**					***0.000
No	611	39.6	31.4	27.9–35.0	
Yes	931	60.4	21.5	18.9–24.3	
**Ate fruits daily**					0.163
No	777	50.4	27.1	24.1–30.3	
Yes	765	49.6	25.6	21.1–27.2	
**Ate vegetables daily**					
No	514	33.3	26.1	22.4–29.9	0.746
Yes	1028	66.7	25.3	22.7–28.1	
**Other tobacco use**					0.320
No	1,450	94.0	25.8	23.6–28.1	
Yes	92	6.0	21.1	13.1–30.8	
**Alcohol use**					0.597
No	1354	87.8	25.3	23.1–27.7	
Yes	188	12.2	27.1	21.2–33.9	
**Bullying victimisation**					**0.028
No	872	59.3	27.7	24.8–30.7	
Yes	670	40.7	22.7	19.7–26.1	
**Sedentary life (time spent sitting)**					0.284
No	1,226	79.5	24.9	22.6–27.4	
Yes	316	20.5	27.8	23.2–33.0	
**Physical Education (PE) class attendance**					0.371
Irregular PE attendance	958	62.1	24.8	22.2–27.6	
Regular PE attendance	584	37.9	26.8	23.4–30.7	
**Active commuting to school**					***0.000
Iregular active Commuting to school	1,066	69.2	19.2	16.9–21.7	
Regular active Commuting to school	476	30.8	39.5	35.2–43.9	
**BMI**					0.732
Underweight	242	15.7	25.9	20.3–31.3	
Normal	1,199	77.8	26.0	23.4–28.4	
Overweight and obese	101	6.6	22.9	15.3–31.4	
**Number of close friends**					0.004
0	200	13.0	23.6	18.2–30.1	
1–2	853	55.3	22.8	20.1–25.8	
3 or more	489	33.7	30.8	26.9–34.9	
**Peer support**					
No	172	11.1	15.8	11.0–22.0	**0.002
Yes	1,370	88.9	26.7	24.5–29.2	
**Parents check homework**					0.204
No	549	35.6	27.4	23.9–31.3	
Yes	993	64.4	24.5	21.9–27.3	
**Parents understands troubles past**					*0.032
No	366	23.7	21.2	17.3–25.8	
Yes	1,176	76.2	26.8	24.4–29.5	
**Parents know what you do past**					0.124
No	440	28.5	28.3	24.2–32.7	
Yes	1,102	71.5	24.5	22.0–27.1	

Computed from GSHS 2012

* p < 0.05

** p < 0.01

*** p < 0.001, CI = Confidence Interval

**Table 2:** Crude Prevalence Ratios (CPR) and Adjusted Prevalence Ratios (APR) of physical activity among SHS students in Ghana.

Variable	Model I CPR (95%CI)	Model II APR (95%CI)
**Demographic**		
**Sex**		
Male	Ref	Ref
Female	0.72***[0.60,0.85]	0.73***[0.61,0.87]
**Grade**		
SHS1	Ref	Ref
SHS2	0.74*[0.57,0.97]	0.75*[0.57,0,98]
SHS3	0.79*[0.64,0.98]	0.80*[0.65,0.99]
SHS4	1.17[0.94,1.45]	1.14[0.91,1.42]
**Socio-economic status (Hunger)**		
No	Ref	Ref
Yes	0.69***[0.58,0.81]	0.71***[0.60,0.84]
**Active commuting to school**		
Iregular active commuting to school		Ref
Regular active commuting to school		1.99***[1.69,2.35]
**Number of close friends**		
0		Ref
1–2		0.93[0.71,1.21]
3 or more		1.15[0.88,1.52]
**Bullying victimization**		
No		Ref
Yes		0.93[0.78,1.11]
**Peer support**		
No		Ref
Yes		1.44*[1.02,2.03]
**Parental bonding**		
No		Ref
Yes		1.12[0.90,1.39]
N	1,542	1,542
pseudo R2	0.019	0.050

* p < 0.05

*** p < 0.001, CI = Confidence Interval

Source: Computed from GSHS (2012) Dataset

### Ethical approval

Prior to the actual data collection, the questionnaire was piloted to ensure adequate comprehension of the survey items. The survey was approved by the Institutional Review Board at Middle Tennessee State University. All ethical procedures outlined by the Ghana Education Service (GES) regarding the engagement of students in survey studies were duly followed. Permissions were also obtained from GES, heads of the selected schools and classroom teachers. Bothe verbal and written consent were obtained from all the students. For minors, consent was obtained from their parents as well. The dataset is freely available for download at: https://www.cdc.gov/gshs/countries/africa/ghana.htm.

## Results

### Descriptive analysis results

#### Prevalence of physical activity

**Fig 1:**
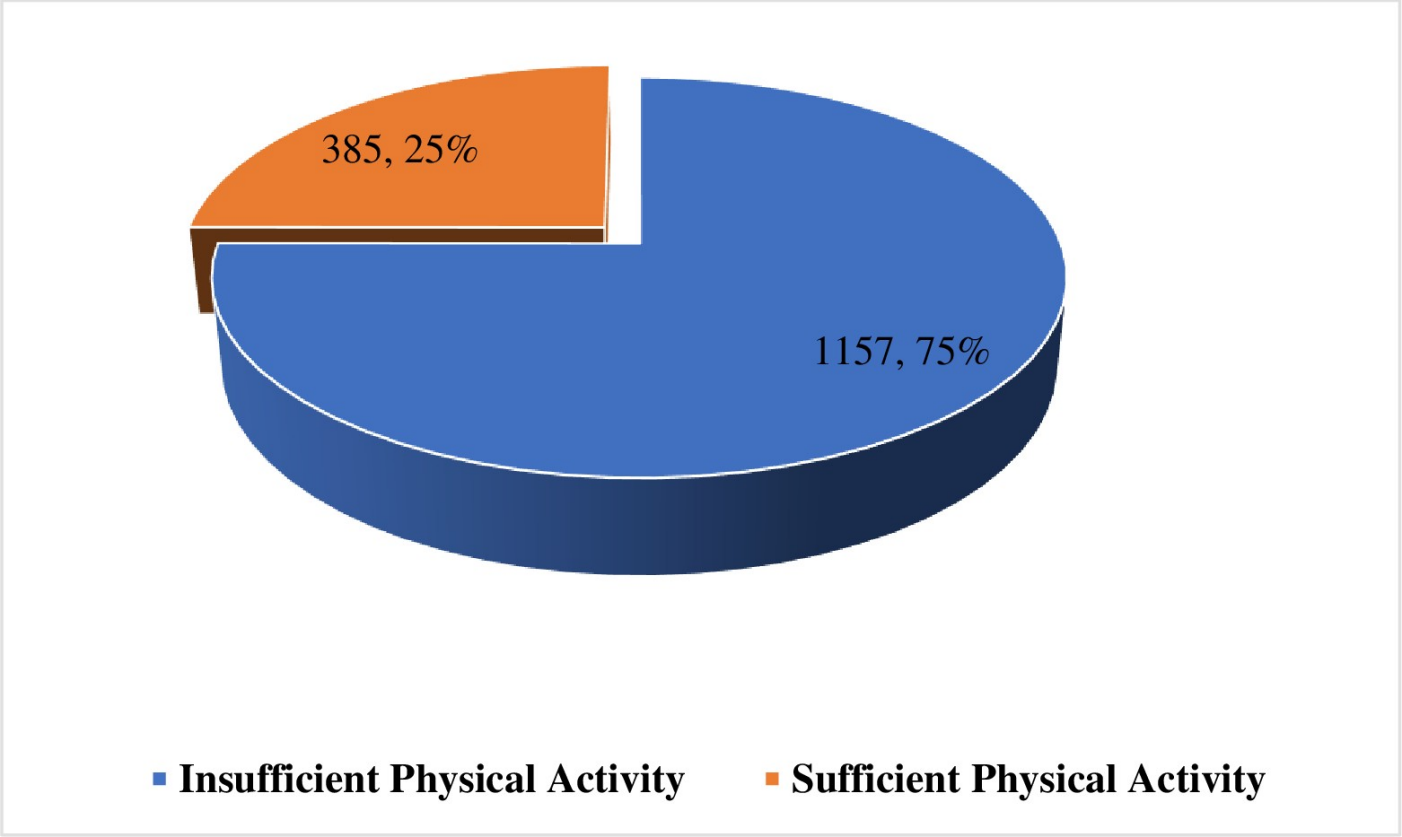
Prevalence of physical activity among SHS students in Ghana.

Of the total 1,542 students who participated in the study, only 25.0% (29.0% males and 21.9% females) were physically active (see Fig 1).

Details of the demographic characteristics of the participants in this study are presented in Table 1. In terms of age, the students aged 18 and above had the highest proportion of sufficient physically active, compared to those aged 12–17. In relation to grade, the students in SHS4 had the highest proportion of sufficient physical activity. It was also shown that students who did not experience hunger—a measure of socio-economic status—had higher proportion of sufficient physical activity compared, to those who experienced hunger. In relation to vegetable and fruit consumption, 26.1% of those who indicated they do not eat fruits and vegetables were physically active. In terms of parental factors, 26.7% of the students who indicated their parents understand their troubles were sufficiently physically active. The chi-square analysis revealed that sex (*χ*^*2*^ = 12.3 p<0.001), grade (*χ*^*2*^ = 14.1, p<0.01), socio-economic status (*χ*^*2*^ = 19.2, p<0.001), bullying victimisation (*χ*^*2*^ = 4.8, p<0.05), active commuting to school (*χ*^*2*^ = 71.5, p<0.001), number of close friends (*χ*^*2*^ = 11.1, p<0.01), peer support (*χ*^*2*^ = 9.6, p<0.01), and parental understanding of troubles (*χ*^*2*^ = 4.6, p<0.05) had statistically significant association with sufficient physical activity (see Table 1).

### Multivariate analysis results

We computed the crude and adjusted prevalence ratios of physical activity to model the association between the independent variables and physical activity. From the multivariate analysis, female students were less likely to report sufficient physical activity, compared to males (APR = 0.73, 95% CI = 0.61, 0.87). Students in SHS2 (APR = 0.75, 95% CI = 0.57, 0.98) and SHS3 (APR = 0.80, 95% CI = 0.65, 0.99) were less likely be physically active, compared to those in SHS1. The adjusted PR of sufficient physical activity was 0.71 among students who went hungry in the past 30 days compared to those who never went hungry (APR = 0.71, 95% CI = 0.601, 0.84). Students who actively commuted to school on regular basis were about 2 times more likely to be physically active compared to those who did not actively commute to school regularly (APR = 1.99, 95% CI = 1.69, 2.35). Students who got support from their peers were more likely to be physically active, compared to those who did not get support from their peers (APR = 1.43, 95% CI = 1.01–2.03) (see Table 2).

## Discussion

This study sought to determine the prevalence and correlates of physical activity among SHS students in Ghana using data from the 2012 version of the GSHS. Of the total 1,542 students who participated in the study, only 25.0% (29.0% males and 21.9% females) met WHO’s recommendations on physical activity. This finding corroborates those of previous studies on adolescent physical activity at the global level [8, 35], in Brazil [11, 17], and in Peru [2]. The proportions of physical activity obtained in this study are higher than what Aguilar-Farias et al. [10] found in Latin America and the Caribbean but lower than what was found in Nigeria [12], Southern Brazil [16], United States of America [36] and the Eastern Mediterranean countries [31]. In addition, the proportion of sufficient physical activity in this study was lower than what was found by Guthold et al. [23] in Ghana. The possible reason for the difference in proportions in these studies might be due to the differences in the approaches used. The possible reason for the low level of physical activity among adolescents could the strong correlation between sedentary behaviour, on the one hand, and the use of computers, televisions and videogames, on the other hand, among Ghanaian adolescents [26]. Another reason could be that some of the students may not have adequate knowledge on physical activity and the health benefits associated with it.

Female students, compared to males, were less likely to report sufficient physical activity, which resonates with findings of some previous studies [17, 31, 37–42]. This finding can also be explained within the context of some social and biological factors [17]. The social explanation, as given by de Lima and Silva [17] and Farias et al. [43], concerns the cultural perception that young girls have fragile body and, therefore, must engage themselves in not-too-forceful activities. Boys, on the other hand, are perceived to have strong bodies and are, therefore, directed to involve themselves in vigorous physical activities. Regarding chances to practice physical activity, these norms could be a disadvantage to girls [43]. In addition, some girls may perceive themselves to suffer negative consequences of participating in physical activities more than boys [18]. It is, therefore, imperative for the government to implement policies aimed at reducing gender differentials relating to physical activity in SHSs. Changes in the family and the school environments, as well as both private and public media, should be considered in the formulation of such policies.

The study further revealed that SHS students from higher socio-economic background (that is, students who do not experience hunger), as opposed to those in lower socio-economic status, have a higher tendency to be physically active. This is consistent with what was found by some previous studies [2, 17, 41, 44]. This could be due to the fact that those who feel hungry may be weak to engage in any vigorous physical activity. Corseuil et al. [45] also explained that the insufficiency in the level of physical activity of students from low socio-economic background could be attributed to the fact that people with low monthly income often live in environments without facilities (such as parks, walking and running trails, and sidewalks) that promote children’s involvement in physical activity. In addition, extracurricular activities at school do not guarantee students’ active involvement in physical activity.

It was also found that there is a higher likelihood for students in SHS2 and SHS3 to be physically active, unlike their counterparts in SHS1. This finding confirms the existence of some relationship between adolescents’ physical activity and educational level, as suggested by some studies [46, 47] which note that there is a higher likelihood for adolescents in lower classes to be physically active. There is evidence suggesting that there is a likelihood for physical activity levels of adolescents to decrease with age [46, 47]. In addition, students at the lower levels in SHSs in Ghana, especially those in boarding schools, are considered as juniors and, for that matter, are directed to do some of the things the seniors (those in SHS2 and SHS3) are supposed to do during and after class. Relatedly, students who had support from their peers had a higher propensity to be physically active. In explaining this, Dumith et al. [16] noted that physical activity is associated with social support but not cognitive performance. While this may be true, further studies using mixed methods approach may be needed to offer a detailed explanation to such a correlation.

Our study also found that the likelihood of being physically active was higher among SHS students who walked to school. This seems to substantiate the view that a decrease in physical activity among adolescents largely results from a decline in the number physical activities but not the duration of such activities [48]. It is, therefore, necessary to encourage SHS students to actively involve themselves in a number of physical activities. It is also important to establish a correlation between engagement in physical activity during adolescence and adulthood [16].

### Strength and limitations

The fact that the present study used a relatively large sample from SHS makes it essentially representative of SHS students in Ghanaian. Despite this strength, the measurement of physical activity using a single-item questionnaire is the main limitation of the study. This is because physical activity could be measured by instruments such as accelerometers or pedometers which offer better insights into different physical activity intensities. It is also necessary to contextualise the occurrence of physical activity among adolescents, as suggested by some researchers [49]. Moreover, the research design (cross-sectional) employed for the data collection does not allow one to conclusively establish causal links between the variables but could only establish associations. Also, there could be recall biases resulting from the data collection method used (that is, self-report responses), which could affect the estimation of physical activity. Finally, the involvement of only SHS students, without considering the out-of-school, is also a limitation of this study which makes generalization to the out of school difficult.

## Conclusion

Only a quarter of SHS students in Ghana met the recommended levels of physical activity by the WHO. Subgroups of SHS students who were less likely to be physically active are females, those in SHS2 and SHS3, and those from low socio-economic background (experienced hunger). However, those who actively commute to school and those who get support from their peers are more likely to be physically active. Physical activity is a major component of any health promotion program and, for that matter, it is necessary to invest in policies on physical activity promotion among females, second and third year SHS students as well as adolescents who struggle to attain the WHO recommended level of physical activity.

## Supporting information

S1 Table (DOCX)

S2 Table (DOCX)
