# Supplementary material for: Are senior high school students in Ghana meeting WHO’s recommended level of physical activity? Evidence from the 2012 Global School-based Student Health Survey Data
Source: PLoS One. 2020 Feb 12;15(2):e0229012. doi: 10.1371/journal.pone.0229012 (PMC7015424; doi:10.1371/journal.pone.0229012)
Supplement: S2 Table — (DOCX) [file pone.0229012.s002.docx]

**S1 Table: Variables descriptions and coding.**

| **Variables** | **Question** | **Original Response Options** | **New code** |
| --- | --- | --- | --- |
| **Outcome variable** | |  |  |
| 1. Physical activity | ﻿﻿During the past 7 days, on how many days were you  physically active for a total of at least 60 minutes per  day? | 1=0 days  2= 1 day  3=2 days  4=3 days  5=4days  6=5days  7=6days  8=7 days | **1-5=“inactive 6-8=“sufficiently physically active”** |
| **Socio-demographic variables** | | |  |
| 1. Age | Custom age | 1=12  2=13  3=14  4=15  5=16  6=17  7=18 years | 1=12-17  2=18 and above |
| 1. Sex | Sex | 1=Male, 2=Female | 1=Male, 2=Female |
| 1. Grade | In what grade are you? | 1=SHS1, 2=SHS2,3=SHS3,4=SHS4 | 1=SHS1, 2=SHS2,3=SHS3,4=SHS4 |
| 1. Hunger (Socio-economic status) | Went hungry past 30 days | 1=never,  2=Rarely,  3=sometimes,  4=most of the times  5=always | 1 -2=No  3-5=Yes |
| 1. Body Mass Index   Height Weight | How tall are you without your shoes on? (Data are in meters.)  How much do you weigh without your shoes on?(Data are in kilograms | | ****See foot note** |
| **Fruits and vegetable consumption** | | |  |
| 1. Fruits | ﻿During the past 30 days, how many times per day did you usually eat fruit, such as oranges, pineapple,  watermelon, banana, guava, pear, sweet apple,  mangoes, or pawpaw? | 1= ﻿I did not eat fruit during the past 30 days  2=﻿Less than one time per day  3=1 time per day  4=2 times per day  5=3 times per day  6=4 times per day  7=5 or more times per day | **1-2 = No**  **3–7 = Yes** |
| 1. Vegetables | ﻿During the past 30 days, how many times per day did  you usually eat vegetables, such as kontomire, garden  eggs, lettuce, cabbage,okra, alefu, bira, ayoyo, or bean leaves? | 1= ﻿I did not eat vegetables during the past 30 days  2=﻿Less than one time per day  3=1 time per day  4=2 times per day  5=3 times per day  6=4 times per day  7=5 or more times per day | **1 -2 = No**  **3–7 = Yes** |
| **Substance use and alcohol use and other behaviour** | | |  |
| 1. Tobacco use | ﻿During the past 30 days, on how many days did you  use any tobacco products other than cigarettes, such as  tawa snuff powder, chewing tobacco, paper rolled  tobacco, dip, cigars, or pipe? | 1 = 0 days  2=1 or 2 days  3=3 to 5 days  4= 6 to 9 days  5=10 to 19 days  6=20 to 29 days  7 = All 30 days | **1 = No**  **2–7 = Yes** |
| 1. Alcohol use | ﻿During the past 30 days, on how many days did you  have at least one drink containing alcohol? | 1 = 0 days  2=1 or 2 days  3=3 to 5 days  4= 6 to 9 days  5=10 to 19 days  6=20 to 29 days  7 = All 30 days | **1 = No**  **2–7 = Yes** |
| 1. Bullying victimisation | ﻿During the past 30 days, on how many days were you  bullied? | 1 = 0 days  2=1 or 2 days  3=3 to 5 days  4= 6 to 9 days  5=10 to 19 days  6=20 to 29 days  7 = All 30 days | **1 = No**  **2–7 = Yes** |
| 1. Engaged in Sedentary behaviour | ﻿How much time do you spend during a typical or usual day sitting and watching television, playing computer  games, talking with friends, or doing other sitting  activities such as Oware, Ludu, Draft, Snake and  Ladders, or other board games? | 1=Less than 1 hour per day,  2=2,1 to 2 hours per day  3=﻿3 to 4 hours per day  4=﻿5 to 6 hours per day  5=﻿7 to 8 hours per day  6 =More than 8 hours per days | **1-3 = No**  **4-5 = Yes** |
| 1. Active commuting to school | ﻿During the past 7 days, on how many days did you  walk or ride a bicycle to/from school? | 1=0 days  2=1 day  3=2 days  4=3 days  5=4 days  6=5 days  7=6days  8=7 days  **(Coded as 1-3= 0, 4-8=1)** | **1-3= Irr**egular Active Commuting to school  **4-8=** Regular Active Commuting to school |
| 1. PE class attendance | ﻿During this school year, on how many days did you go  to physical education (PE) class each week? | 1=0 days  2=1 day  3=2 days  4=3 days  5=4 days  6=5 or more days | **1-3= Irr**egular PE attendance  **4-8=** Regular PE attendance |
| **Socio-familial support** | | |  |
| 1. Helpful (Peer support) | ﻿During the past 30 days, how often were most of the  students in your school kind and helpful? | 1 = never  2=rarely  3=sometimes  4=most of the time  5 = always | **1-2= No**  **3-5=Yes** |
| 1. Parents check homework (parental supervision) | ﻿During the past 30 days, how often did your parents or  guardians check to see if your homework was done? | 1 = never  2=rarely  3=sometimes  4=most of the time  5 = always | **1-2= No**  **3-5=Yes** |
| 1. Understand problems (Parental connectedness | ﻿During the past 30 days, how often did your parents or  guardians understand your problems and worries? | ﻿1 = never  2=rarely  3=sometimes  4=most of the time  5 = always | **1-2= No**  **3-5=Yes** |
| 1. Know what adolescent do free time (﻿Parental or guardian Bonding) | ﻿During the past 30 days, how often did your parents  or guardians really know what you were doing with  your free time? | ﻿1 = never  2=rarely  3=sometimes  4=most of the time  5 = always | **1-2= No**  **3-5=Yes** |

**BMI was calculated as weight (kg)/height (m ^2^) and classified as follows: <18.5 kg/m2, underweight; 18.5–24.9 kg/m2, normal; 25.0–29.9 kg/m2, overweight; and >29.9 kg/m2, obesity using WHO, 2015 reference standards)
